# Bioinspired Hydrogel Coating Based on Methacryloyl Gelatin Bioactivates Polypropylene Meshes for Abdominal Wall Repair

**DOI:** 10.3390/polym12081677

**Published:** 2020-07-28

**Authors:** Andrada Serafim, Sergiu Cecoltan, Elena Olăreț, Diana-Maria Dragusin, Eugeniu Vasile, Valentin Popescu, Bogdan Stelian Manolescu Mastalier, Horia Iovu, Izabela-Cristina Stancu

**Affiliations:** 1 Advanced Polymer Materials Group, University Politehnica of Bucharest, 1-7 Gh. Polizu Street, 011061 Bucharest, Romania; andrada.serafim@gmail.com (A.S.); sergiu.cecoltan@gmail.com (S.C.); olaretelena@gmail.com (E.O.); diana.m.dragusin@gmail.com (D.-M.D.); horia.iovu@upb.ro (H.I.); 2 Department of Science and Engineering of Oxide Materials and Nanomaterials, University Politehnica of Bucharest, 1-7 Gh. Polizu Street, 011061 Bucharest, Romania; eugeniuvasile@yahoo.com; 3 Department of General Surgery, Colentina Clinical Hospital, 19–21 Stefan cel Mare, 72202 Bucharest, Romania; popescu.vali.UMF@gmail.com (V.P.); bogdanmastalier@yahoo.com (B.S.M.M.)

**Keywords:** methacryloyl gelatin, methacryloyl mucin, polypropylene mesh, bioinspired hydrogel coating, PRP treatment

## Abstract

Considering the potential of hydrogels to mimic the cellular microenvironment, methacryloyl gelatin (GelMA) and methacryloyl mucin (MuMA) were selected and compared as bioinspired coatings for commercially available polypropylene (PP) meshes for ventral hernia repair. Thin, elastic hydrated hydrogel layers were obtained through network-forming photo-polymerization, after immobilization of derivatives on the surface of the PP fibers. Fourier transform infrared spectroscopy (FTIR) proved the successful coating while the surface morphology and homogeneity were investigated by scanning electron microscopy (SEM) and micro-computed tomography (micro-CT). The stability of the hydrogel layers was evaluated through biodynamic tests performed on the coated meshes for seven days, followed by inspection of surface morphology through SEM and micro-CT. Taking into account that platelet-rich plasma (PRP) may improve healing due to its high concentration of growth factors, this extract was used as pre-treatment for the hydrogel coating to additionally stimulate cell interactions. The performed advanced characterization proved that GelMA and MuMA coatings can modulate fibroblasts response on PP meshes, either as such or supplemented with PRP extract as a blood-derived bioactivator. GelMA supported the best cellular response. These findings may extend the applicative potential of functionalized gelatin opening a new path on the research and engineering of a new generation of bioactive meshes.

## 1. Introduction

With over 1 million repair interventions performed annually worldwide, abdominal hernia represents one of the most common surgical problems [1,2]. Polypropylene was introduced as a biomaterial in the 1960s and since then other polymers became available for hernia repair (e.g., polytetraflourethylene, polyurethane, polyester, polyethylene etc.) [3,4,5,6]. Although regarded as golden standard for tissue reinforcement, implantation of a synthetic mesh usually generates a foreign body response from the host which can lead to the formation of scar tissue, seroma or tissue degradation followed by chronic pain and discomfort [1,2,6,7]. Extensive efforts have been devoted to improve performances; therefore, more than 70 types of meshes for hernia repair are commercially available now [8]. Three generations of products were developed: synthetic non-absorbable meshes, mixed or composite implants and biological prostheses. Having a high rate of integration, the bio-meshes promote the rebuilding of the natural tissue, but their mechanical properties are not as robust as the PP meshes [6]. Added value can be provided by coating the PP meshes [8]. While the synthetic frame provides the optimal mechanical properties, a naturally-derived coating or side layer might promote enhanced tissue integration. Parietex for example, the first composite product fabricated from polyester and collagen, which acts as a barrier on the visceral side, leads to enhanced in vitro cellular proliferation when compared to the control unmodified PP mesh [4]. Investigated coatings include polymers such as polylactic and polyglycolic acids, poly(n-vinyl pyrrolidone), cellulose-based materials, collagen and chitosan. Hydrogel coatings based on naturally-occurring polymers, chemically attached to a substrate represent an appealing route of providing a stable biocompatible and cell-interactive surface [9]. Such hydrated microenvironments recalling the properties of the extracellular matrix (ECM) are often appealing for additional loading with bioactive molecules such as antibiotics [10], antimicrobial agents [11] or growth factors [12] to further enhance the integration phenomena. Given the fact that fibroblasts are the main cells responsible for the synthesis of ECM and collagen, accordingly mediating the wound healing in mesh reinforcing, methods to control their proliferation are also required. Plated-rich plasma (PRP) is an autologous concentrate with a demonstrated ability of improving the healing process due to the high content of natural growth factors [13]. While improved fibroblasts attachment has been reported on PRP-treated meshes [14], the use of PRP remains yet underexplored in this type of applications. A recent review [14] gives an overview on reported findings and limitations associated with the use of PRP. In the present work we investigate the potential of two hydrogel coatings combined with a PRP pre-incubation treatment to stimulate fibroblasts interactions with PP meshes. Methacryloyl derivatives of proteins, and particularly methacryloyl functionalized gelatins, are increasingly used in regenerative medicine and tissue engineering applications due to their ability to be further polymerized while maintaining the biocompatibility of the pristine protein. Our group has previously used methacryloyl gelatin (GelMA) to improve cell interactivity of synthetic polymers for tissue regeneration [15,16], while methacryloyl mucin (MuMA) also leads to stable hydrogels and has been investigated for controlled release of active molecules [17,18]. Based on our previous experience in preparing GelMA and MuMA scaffolds, we believe that they also have potential for reconstructive medicine; therefore, in this study, our aim was to engineer ECM-bioinspired coatings for PP meshes and further explore their potential in improving the efficiency of PRP in promoting fibroblasts interactions.

## 2. Materials and Methods

### 2.1. Materials

Unless otherwise stated, all materials employed in this study were purchased from Sigma-Aldrich and used without any further purification.

### 2.2. Methods

#### 2.2.1. Preparation of Hydrogel Coatings

Commercially available polypropylene (PP) meshes (HERNI PRO, type P3, Biosintex) were used as a model in this study. Methacryloyl derivatives of gelatin (GelMA) and mucin (MuMA) were used to coat the meshes. MuMA was obtained from porcine gastric mucin as previously described in [17], using a weight ratio of mucin: methacrylic anhydride of 20:1. GelMA was also obtained through the direct reaction of cold water fish gelatin with the methacrylic anhydride at a weight ratio of 1:3 following the protocol reported in [15]. The coating procedure was developed as a three-step process. First, the PP meshes (intensively washed with Tween 80 and ethanol using an ultrasound bath) were submitted to plasma activation with carbon dioxide (CO_2_) using a FEMTO type C device (Diener electronic) at 100 W for 1 min on each side. The surface functionalization with GelMA and MuMA was performed after activation of generated -COOH groups with 1-ethyl-3-(3-dimethylaminopropyl)carbodiimide hydrochloride (EDC) (0.4 mg/mg PP) and N-hydroxysulfosuccinimide (NHS) (Fluka) (0.6 mg/mg PP) in 0.1 M [2-(N-morpholino)ethane sulfonic acid] (MES) containing NaCl (0.5 M), pH 6.0. The samples were immersed in a protein derivative solution (50 mg/mL) containing Irgacure 2959 (1:1000 weight ratio with respect to protein). The meshes were rinsed with Tris buffer to remove physically retained protein, then submitted to photopolymerization using a transilluminator ECX-F26, at 312 nm and immersed in double distilled water to remove unreacted molecules.

#### 2.2.2. Characterization of Coating

The success of the coating process was investigated by different tests on modified meshes. Pristine meshes were used as control. Having a low contrast between the synthetic polymer core and the natural polymer based coating, the coated samples were treated with silver nitrate (1% AgNO_3_ aqueous solution for 1 h under direct light) [14]. Scanning electron microscopy (SEM) and micro-computed tomography (micro-CT) imaging were employed to visualize the protein coatings.

##### Attenuated Total Reflectance-Fourier Transform Infrared Spectroscopy (ATR-FTIR)

ATR-FTIR spectra were collected using a spectrometer JASCO 4200 equipped with a Specac Golden Gate attenuated total reflectance (ATR) device, using a resolution of 4 cm^−1^ and an accumulation of 250 spectra in the wavenumber range of 4000–600 cm^−1^.

##### Contact Angle (CA) Measurements

Samples were subjected to CA measurements throughout the different stages of the coating experiment. In this respect, the following meshes were tested: (a) PP mesh (PP0), (b) PP mesh post plasma treatment (plasma-PP), (c) GelMA-coated PP mesh (PPG), and (d) MuMA-coated PP mesh (PPM). The CA measurements were conducted using a Drop Shape Analyzer DSA 100 (Kruss, Germany) by dropping 1.5 µL onto PP meshes in the knot area, using a needle with a diameter of 0.51 mm. The spreading of the droplet was observed using a video camera with a speed of 10 frames per seconds. The values were averaged over 10 readings in 3 measurements, using Young–Laplace fitting.

##### Stability in Physiologically Simulated Conditions

The stability of the coatings was evaluated trough cyclic traction using the BioDynamic 5210 equipment (Bose Corp., ElectroForce System Group). In this respect, protein-coated PP samples (7 × 50 mm) were fixed in the traction grips inside the testing chambers which were filled with PBS (pH 7.4) using a peristaltic pump (flow 2 mL/min). The tests were performed at a frequency of 1 Hz and a displacement of ±1.5 mm. The mechanical strain was applied for 7 days at 37 °C. At the end of the test the meshes were gently washed with double distilled water and dried at room temperature. The samples were stained, the middle area of the samples was cut, and changes in the morphological aspects and coating homogeneity were investigated through micro-CT and SEM.

#### 2.2.3. PRP Preparation

Whole blood from a healthy donor was drawn in 10 mL T-LAB PRP vacutainers containing 1 mL sodium citrate 0.109 M using a 21 G needle and subsequently centrifuged at 2000 G for 12 min. The procedure was approved by the Ethical Committee of Colentina University Hospital (approval No. 15/13.07.2017). Using the long needle, PRP was transferred into the re-suspension tube and gently stirred for about 1 min. The obtained solution was lysed on the ultrasound bath for 5 min and then centrifuged at 3300 G for 7 min. The platelet count showed that the process resulted in a fivefold platelet concentration when compared with the donor whole blood and further used in a concentration of 4× in Dulbecco’s Modified Eagle Medium (DMEM) for the bioactivation of the hydrogel coatings.

#### 2.2.4. In Vitro Biocompatibility

Murine fibroblast cell line L-929 was cultivated in DMEM supplemented with 10% fetal bovine serum (FBS), penicillin/streptomycin. The hydrogel-coated PP meshes were mounted in 96-wells ultra-low attachment plates (Costar 3474) and incubated for 2 h at 37 °C with 30 µL PRP medium prepared as described at 2.2.3. Pristine PP meshes were used as controls. The samples were washed twice with DMEM media and seeded with L-929 fibroblast cells at a concentration of 1 × 10^4^ cells/well, 2.5 × 10^3^ cells/well, and 1 × 10^3^ cells/well for 1, 3, and 7 days, respectively, and incubated in a humidified atmosphere with 5% CO_2_ at 37 °C. Fibroblasts adhesion was explored at 1 day post-seeding, while proliferation was assessed at 3 and 7 days post-culture. The non-adherent cells were removed after one day; the medium was changed every 48 h throughout the experiment. Cells were seeded on PP mesh, hydrogel-coated PP mesh (denoted PPG and PPM, respectively), PRP-bioactivated PP mesh (denoted PPP), and PRP-bioactivated hydrogel-coated PP mesh (denoted PPGP and PPMP, respectively). To facilitate understanding, the post-seeding time is indicated by _1, _3 and _7 in the samples code, where 1, 3 and 7 represent the number of days post-cells seeding. Cells viability was estimated by quantifying the total lactate dehydrogenase (LDH) at 1, 3, and 7 days respectively, using LDH kit TOX7 (Sigma). The absorbance values for samples were normalized to those obtained for cells cultured in the same conditions on pristine PP as control. Statistical significance for difference between groups was assessed using the unpaired, two-tailed student *T*-test in R. For all imaging analysis, the seeded samples were fixed in 4% paraformaldehyde in PBS for 2 h and then washed with PBS. The samples were permeabilized with 0.1% Triton X-100 (Merck) for 15 min and washed with PBS. To obtain a better imaging through fluorescence microscopy, the seeded samples were stained with DAPI and Phalloidin-TRICT and subsequently mounted in glycerol to reduce PP refraction. The analysis was performed using a Zeiss Axiovert A1-FL-led microscope equipped with an Axio 503m monochrome camera. Cells’ morphology and spreading on the modified scaffolds were assessed by SEM and micro-CT as described in Section 2.2.5 and Section 2.2.6.

#### 2.2.5. Scanning Electron Microscopy (SEM)

The study was performed using a QUANTA INSPECT F SEM device equipped with a field emission gun (FEG) with a 1.2 nm resolution and an X-ray energy dispersive spectrometer (EDS). The SEM images performed to visualize the protein coatings were registered both in back-scattered electron (BSED) and Everhart–Thornley detector (ETD) mode. For better visualization in BSED, Ag-staining was performed. For the investigation of cells’ morphology and spreading, the samples were dehydrated using a gradient of ethanol. All samples were coated with a thin layer of gold before analysis.

#### 2.2.6. Micro-Computed Tomography (micro-CT)

Coated meshes were scanned using a SkyScan 1272 high-resolution X-ray microtomograph (Bruker Micro-CT, Belgium) using the control software version 2.3.0. The recorded 2D projections were processed using CT NRecon (version 1.7.1.6) software and 3D reconstructed using CTVox (version 3.3.0r1403). The homogeneity of the coatings was evaluated using an accelerating voltage of 45 kV and a beam current of 200 µA with no filter during scanning. The acquisition time was 550–600 ms. The pixel size was fixed at 2.75 µm and the rotation step was set at 0.1 degrees.

The L929-seeded samples were scanned at a tension of 70 kV and a beam current of 130 µA. A 0.25 aluminum filter was used. The acquisition time was 3313 ms. The pixel size was 1.5 µm and the rotation step 0.3 degrees. Prior to scanning the samples were kept for one hour in 0.5% uranyl acetate aqueous solution, washed intensively with double distilled water and dehydrated using a gradient of ethanol. The opacity channel was activated in CTVox and adjusted to isolate the natural-derived coating from the PP mesh and imagine it separately. The same procedure was also used in the case of cell-seeded hydrogel-coated meshes.

## 3. Results

Two new types of coatings for PP meshes for hernia repair were developed to improve fibroblasts interactions. PRP was used to supplementary bioactivate the meshes.

### 3.1. Characterization of Hydrogel Coatings

**Figure 1:**
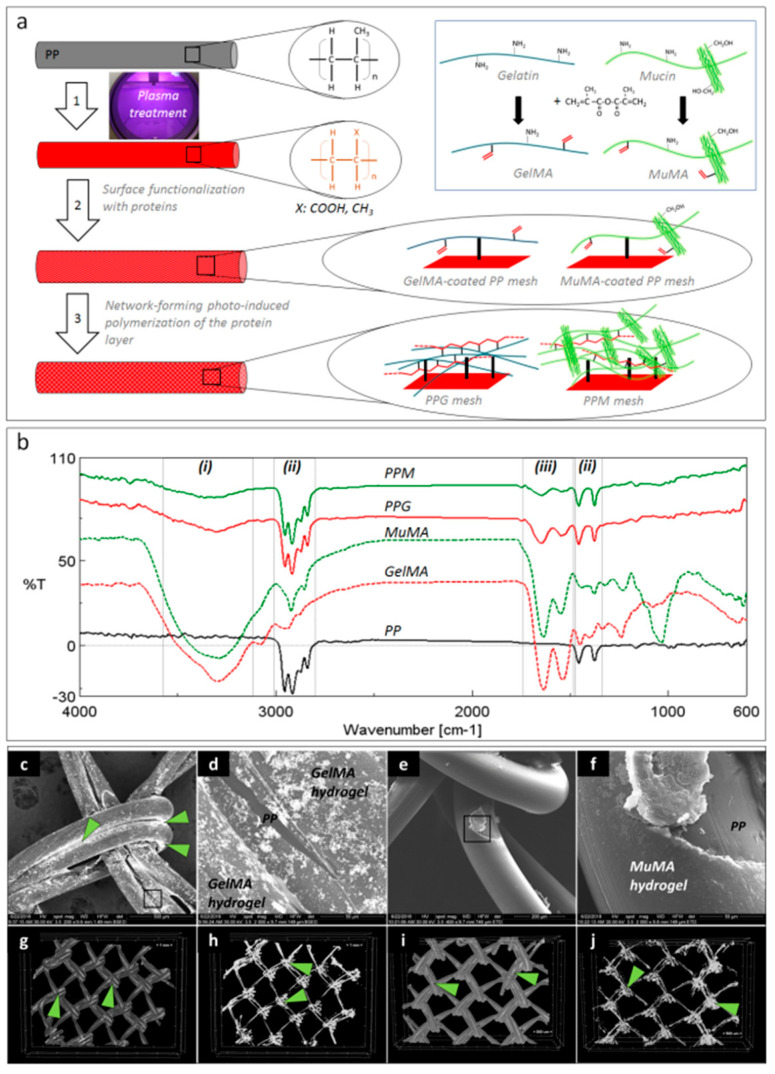
Coatings’ characterization. (**a**): Schematic representation of the three-step procedure used for the surface functionalization of PP meshes: 1—plasma treatment, 2—functionalization with protein analogues GelMA and MuMA (synthesis in inset), 3—generation of polymer-based hydrogels as coatings for the PP fibers; (**b**): FTIR spectroscopy (ATR): solid black—unmodified PP, dashed red and green—methacryloyl derivatives of gelatin and mucin, GelMA and MuMA, solid red and green—hydrogel-coated meshes, PPG and PPM with characteristic peaks for proteins (i and iii) and PP (ii); (**c**–**f**): SEM micrographs demonstrating the homogeneity of the hydrogel coating; (**c**): PP fibers are coated with GelMA, Ag-staining is visible under BSED mode; (**d**): microstructural detail corresponding to the selected area in panel **c**, GelMA hydrogel uniformly covers one PP fiber, while PP substrate is visible in a hydrogel surface fracture (BSED mode); (**e**): MuMA hydrogel uniform coatings at the surface of PP fibers (ETD mode); (**f**): microstructural detail corresponding to the selected area in panel **e** (ETD); (**g**–**j**): micro-CT images: green arrows point Ag agglomerations concentrated at the nodes of the mesh ((**h**,**j**)—micro-CT images of the isolated protein coating).

Figure 1a depicts the schematic representation of the three-step surface modification procedure applied for coating the PP surface with GelMA and MuMA hydrogels. The plasma treatment generated oxygen-containing functional groups (carboxyl and hydroxyl) on the PP meshes, as reported in [19]. A zero-length crosslinking system (EDC/NHS) was used to chemically attach the protein of interest onto the PP surface, while a stable layer of hydrogel was expected to form through free radical polymerization of C=C bonds from methacryloyl groups using photo-initiation. The presence of the protein coating on the PP meshes was proved by ATR-FTIR (Figure 1b). Spectra were recorded on randomly selected areas on the surface of coated meshes (PPG and PPM) using pristine PP, GelMA and MuMA hydrogels as controls. Both PPG and PPM meshes, in addition to characteristic vibrations of PP (ii regions in Figure 1b: strong stretching of C-H groups in the range 3000–2800 cm^−1^ and deformation of C-H at 1456 and 1375 cm^−1^) displayed vibrations specific to GelMA and MuMA (i region in Figure 1b with a broad and strong peak around 3300 cm^−1^ assigned to overlapping O–H and N–H stretching in proteins, and iii region in Figure 1b with amide I and II peaks around 1630 cm^−1^ and 1540 cm^−1^).

No obvious macroscopic modifications of the geometrical/architectural features of the meshes were noticed. Changes in the surface morphology, microstructure and hydrophilic character of the PP meshes after the treatment with proteins were expected to appear and represented other essential aspects in establishing the successful generation of homogeneous coatings. The hydrogels are amorphous and hydrophilic while the synthetic core fiber is semi-crystalline and hydrophobic. SEM micrographs in BSED mode successfully identified Ag nanoparticles agglomerated onto the surface of the meshes with increasing concentration at the interfacial area between two adjacent fibers and knot area coated by proteins (Figure 1c,d). Silver did not stain the PP substrate, as can be noticed in a surface defect resulted into the fractured hydrogel (Figure 1d). Such defects are assigned to the shrinkage of the protein layer at dehydration during sample preparation, while the PP core is hydrophobic and can be noticed under the fractured hydrogel coating (Figure 1d). SEM micrographs in ETD mode revealed a new amorphous layer on top of the PP fibers, suggesting homogeneous coating with proteins. Again, surface fractures suggest the hydrophilic nature of the superficial layer (Figure 1f). While SEM allows a 2D investigation of the coating homogeneity, the micro-CT confirms its uniform distribution (Figure 1g–j) and, again, silver’s tendency to agglomerate in the knots area.

CA measurements were performed on pristine meshes (PP0: 105.44° ± 6.50°), after plasma treatment (plasma-PP: 16.46° ± 0.09°), and after coating with GelMA (PPG: 88.38° ± 4.59°) and MuMA (PPM: 32.17° ± 4.30°), respectively. Plasma treatment influenced the wettability of the mesh through generation of hydrophilic groups (i.e., COOH and OH) on the surface, leading to a significantly decreased CA value. After treatment with the two protein derivatives, the hydrophilic nature of the surface is confirmed through values smaller than 90°. Moreover, the highly hydrophilic nature of native mucin, due to the presence of a high number of hydroxyl groups, was maintained in the MuMA derivative, leading to a CA of PPM considerably lower than the one registered for the PPG.

**Figure 2:**
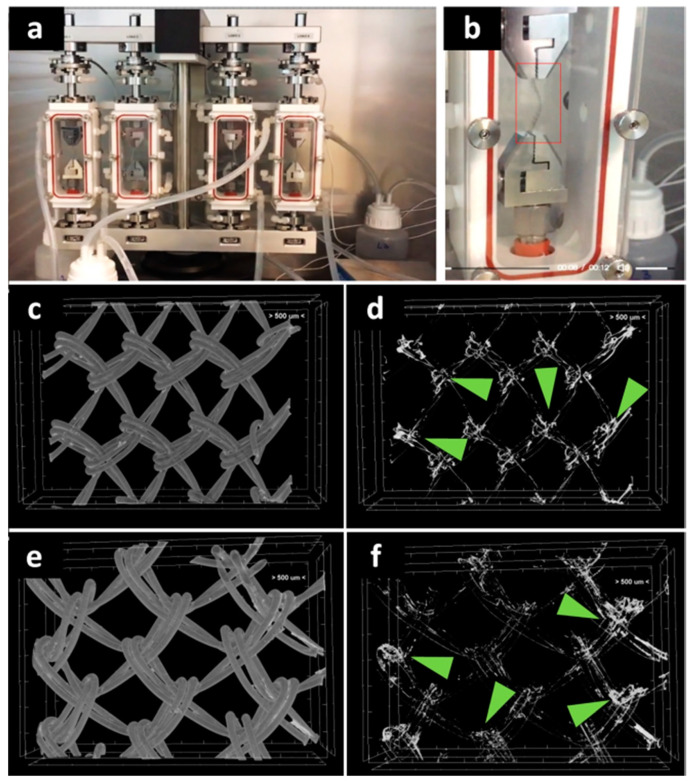
Investigation of protein coating stability at seven days through biodynamic testing: digital photographs of meshes during testing (**a**,**b**). Micro-CT images of the protein-coated PP meshes after seven days biodynamic test: (**c**) PPG; (**d**) GelMA coating after isolation of the natural-derived coating from the PP mesh; (**e**) PPM; (**f**) MuMA coating isolated from the PP mesh; green arrows indicate silver (staining the hydrogel) richer areas at the mesh knots.

Given the importance of the first seven days post-surgery on the new tissue formation on PP meshes [20], it became important to investigate the stability of the hydrogel coating at seven days of dynamic uniaxial traction under PBS perfusion at constant flow (Figure 2a,b). The micro-CT imaging of the middle area revealed a relatively homogeneous coating (Figure 2c,e), while the silver staining is agglomerated in the knots area (Figure 2d,f). This proves that the protein is still coating the fibers at the end of the seven days.

### 3.2. Effect of Approached Route on PP Bioactivation

**Figure 3:**
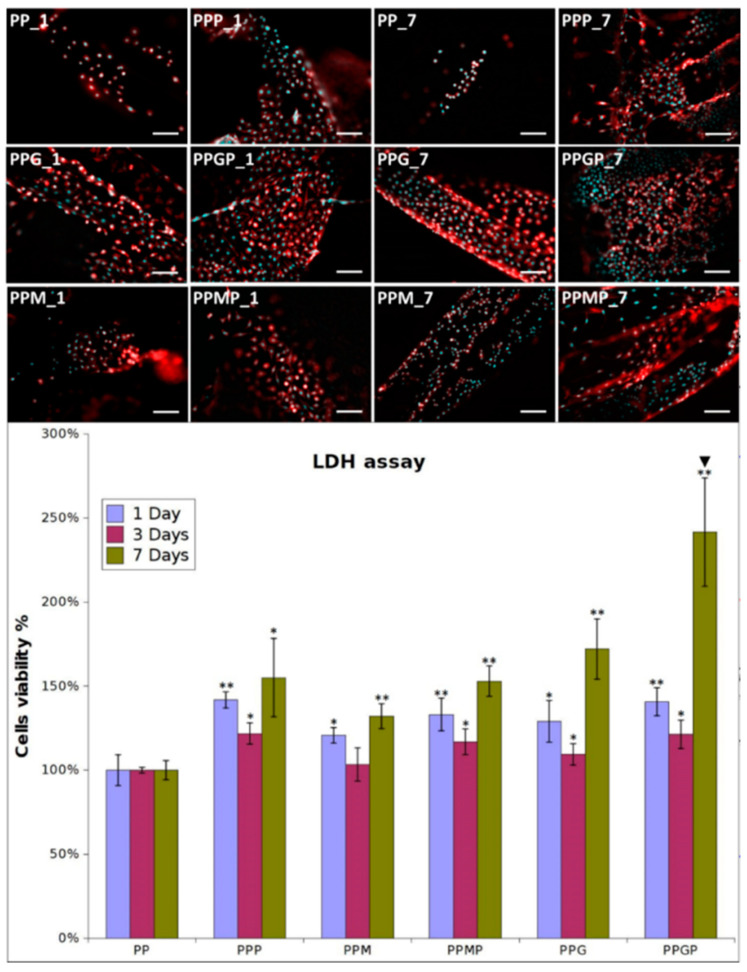
Representative optical microscopy images of fibroblasts on coated meshes at one and seven days post-culture (nuclei stained with DAPI, cytoskeleton stained with Phalloidin-TRICT; magnification 20×, scale bar 100 µm); LDH assay at one, three and seven days post-seeding (*n* = 3, * *p* ≤ 0.05, ** *p* ≤ 0.01, ▼ *p* ≤ 0.01 for PPP vs. PPMP and PPGP, respectively); note that different cell density was used at seeding for the three experimental times.

Fibroblasts were seeded in culture media on pristine PP and hydrogel-coated PP meshes, as such and after PRP treatment. They excellently adhered and proliferated on the coated model implants; adhesion was explored at one day post-seeding, while proliferation was assessed at three and seven days post-culture. Figure 3 shows the optical micrographs for all the tested samples at one and seven days. The cells adhered on the PPG_1 and PPM_1 presented more elongated morphology, indicating improved interaction with the filaments, while those seeded on PP_1 have a more spheroidal morphology typical to poorly adhered cells. A higher number of cells cover the meshes when hydrogels are used as coatings. The LDH results confirmed this observation (Figure 3 samples PP_1, PPM_1, and PPG_1), suggesting that as early as one day post-culture, the two coatings support enhanced fibroblasts adhesion. The type of hydrogel influenced the cell adhesion, with more elongated morphology and better attachment on PPG_1 when compared to PPM_1. Given the fact that the non-adhered cells were removed after 48 h post-seeding, the quantification of viable cells indicated that after three days an early proliferation was noticed on the coated substrates, more intense on GelMA-modified surfaces. After seven days, the fibroblasts excellently proliferate on PPG_7 and PPM_7, reaching confluence on the modified filaments. The morphology of cells suggests a better adhesion on the sample coated with GelMA than on the MuMA-coated PP. According to the LDH results (Figure 3), the protein coatings stimulate proliferation, with the best results obtained for GelMA (PPG_1, _3, and _7). Interactions with fibroblasts are more efficiently stimulated when PRP treatment is performed as evidenced by the morphological features and number of L929 cells (Figure 3). After one day, the number of cells on PPP is higher than in the absence of PRP and their morphology is combined, spheroidal and elongated. Adding PRP on GelMA- and MuMA-coated meshes further enhanced the stimulating effect of the hydrogels on cell adherence and proliferation. According to Figure 3, the best results are obtained for PRP-treated GelMA-coated meshes at all three experimental time points. Early proliferation was noticed at three days and excellent proliferation occurred at seven days.

**Figure 4:**
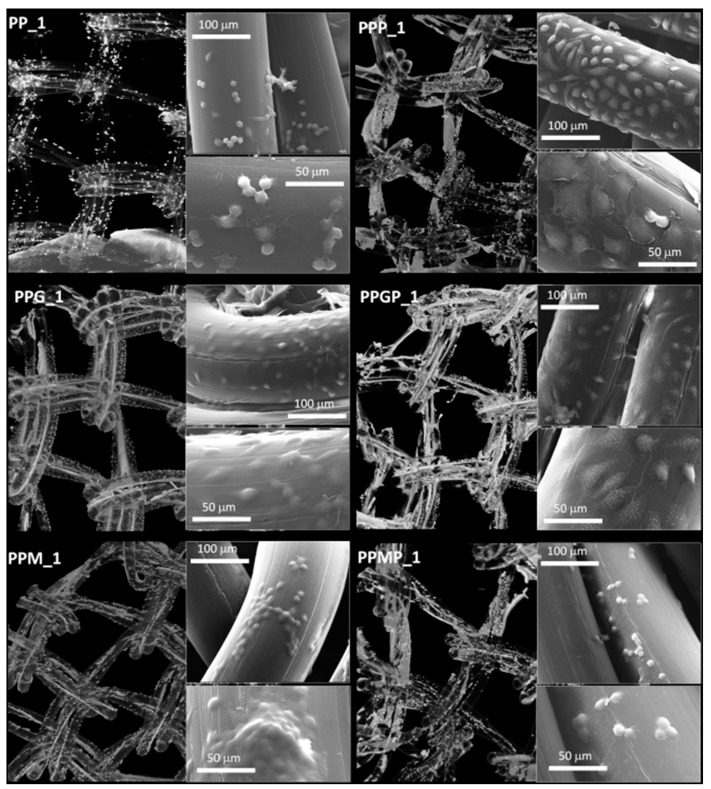
Micro-CT images and SEM micrographs (insets) at one day post-seeding; left column, top to bottom: PP_1 and protein-coated PP (PPG_1 and PPM_1); right column, top to bottom—PRP-treated PP (PPP_1) and PRP-treated hydrogel-coated sample PPGP_1 and PPMP_1.

**Figure 5:**
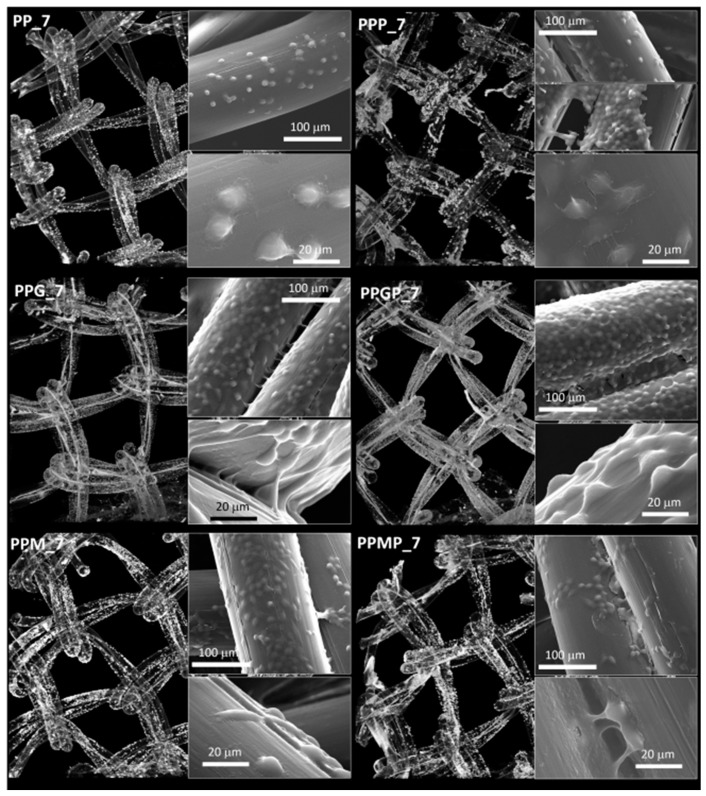
Micro-CT images and SEM micrographs (inset) at seven days post-seeding; left column, top to bottom—PP_7 and protein-coated PP (PPG_7 and PPM_7); right column, top to bottom—PRP-treated PP (PPP_7) and PRP-treated hydrogel-coated sample PPGP_7 and PPMP_7.

For a better understanding of the cell-biomaterial interactions, additional information on the distribution and morphology of fibroblasts on the coated meshes was obtained by a combination of micro-CT and SEM investigation, as can be noticed in Figure 4, Figure 5 and Supplementary Figure S1. Micro-CT images describe the effect of the coating type on the 3D distribution of cells, while more detailed morphological features of cells are provided by SEM micrographs of the PP meshes coated with protein derivatives and additionally treated with PRP. Pristine PP meshes were used as control. Micro-CT monitoring allowed to simultaneously observing cell distribution and hydrogel coating after both cells and hydrogels were correspondingly stained, as described in Section 2.2.2 and Section 2.2.6, respectively. The possibility to isolate the naturally derived coating from the PP mesh through varying the opacity of the registered imagines allows identifying only cells and natural products while the PP filaments are virtually extracted. The non-modified PP mesh appeared as transparent and fibroblasts are visible as white dots distributed on the whole surface of the mesh (PP_1 in Figure 4). Increasing the incubation time leads to a denser cell coating after seven days (PP_7 in Figure 5), while the PP mesh remains transparent since no natural compounds are deposited on its surface. For the hydrogel-coated meshes, an organic layer and cells can be noticed at their surface. All the coatings have a certain degree of heterogeneity (Figure 4, Figure S1 and Figure 5), most probably due to shrinkage of the hydrogel layer at dehydration. PRP treatment on PP filaments leads to a natural discontinuous layer visible in PPP_1 (Figure 4) and PPP_7 (Figure 5). Given this behavior, we assume that the naturally derived material visible on the samples treated both with hydrogel and PRP are the result of PRP deposition on the protein coatings. The smallest number of fibroblasts was registered on the control PP mesh. The cell density and the homogeneity of cell spreading on the mesh increased when GelMA and MuMA were deposited on PP, and the treatment with PRP additionally improved cell adhesion (Figure 4, Figure 5 and Figure S1). As depicted in Figure 4, the best adhesion occurred on GelMA coated samples (PPG_1), and after PRP addition, the cells adhered even stronger on both hydrogel layers, with better results on PPGP_1. The combined coating and increased incubation time enhanced the cellular response as noticed in Figure S1 and Figure 5. SEM micrographs provided details regarding the attachment of cells on the surface of the material. Figure 3 shows that the cells seeded on the PP_1 have rounded morphology and seem to lack attachment. The addition of PRP (PPP_1) leads to more elongated cells. GelMA coating also shows better adherence and spreading of the seeded cells (PPG_1), which improves even more once the autologous extract is added (PPGP_1). The cells behavior is not the same for MuMA coating, the fibroblasts being less elongated on PPM_1 and PPMP_1. Moreover, for PP_1 and PPM_1, cell spreading is different when compared to PPG_1 and the PRP treated surfaces, the cells being organized in clusters, indicating a lower affinity for the substrate. The addition of PRP on MuMA-coated meshes does not improve the spreading or the morphology of L929 cells considerably. The morphology of the cells at three days post seeding is presented in Figure S1. The round morphology of cells seeded on untreated PP mesh is maintained throughout the entire study. In contrast, prolonging the test to seven days (Figure 4) increases the number of cells on the treated meshes. While on PP_7 the number of cells does not seem to have increased considerably, on PPP_7 the cells are clearly more numerous and have the typical fusiform morphology of fibroblasts. Better results are registered for the hydrogel-coated samples: both PPG_7 and PPM_7 show numerous cells with elongated shapes. The best results were registered for PPGP_7, on which the cells almost completely cover the surface of the treated mesh. The evaluation performed through SEM and micro-CT is confirmed by the optical microscopy images and LDH assay. At one day post-seeding, the addition of hydrogel leads to a similar cellular response for both formulations (GelMA and MuMA), but at seven days there is a clear increase in the number of cells seeded on GelMA when compared to the ones seeded on MuMA-coated PP. At any time point, the number of cells is bigger for the treated meshes (either with PRP, hydrogel, or PRP and hydrogel) than on pristine PP mesh. In agreement with the SEM micrographs, the best results at seven days post-seeding are offered by the combined treatment with GelMA and PRP.

## 4. Discussion

Abdominal wall repair represents one of the most common surgical procedures and, according to the FDA, the rate of recurrence is considerably lower when a surgical mesh is used [21]. In a recent and comprehensive review, PP is still regarded as the “golden standard” in repairing the herniated abdominal wall [8,22], but due to the high number of complications that may arise following implantation, it cannot be stated that the material is ideal for such an application [22,23]. Various strategies to modify the surface of the PP mesh can improve biointegration [5,20,24]. To this end natural materials (such as chitosan [25], dermal ECM [6], collagen [26,27] and gelatin [22]) have been used, either by embedding the PP mesh [28] or by chemically attachment using an intermediary such as dopamine [29] or cyclodextrine [30]. Furthermore, various active molecules such as antibiotics [31], antimicrobial agents [32] and growth factors [33] have been used in order to reduce the inflammatory response or to speed up the healing process. Despite extensive efforts, there is currently no standard approach to enhance the cellular interactivity of PP meshes for superior tissue integration; it is accepted that modification strategies with a wide range of hydrophilic substances may improve the characteristics of pristine scaffolds in terms of proliferation rate and fibroblasts coating [34]. In the present work, two types of biomimetic coatings with the potential to control cell interactivity of PP meshes were developed. GelMA and MuMA hydrogel coatings were deemed appropriate to mimic ECM features at the surface of the mesh and to mediate fibroblasts adhesion and proliferation before and after PRP treatment. These hydrogels were grafted to plasma activated PP using a well-known EDC-NHS chemistry. Two visualization methods were entailed in imaging the natural polymer coatings, either treated with PRP or not, before and after cell seeding: SEM and micro-CT. The synergy of these techniques offered valuable insights regarding the efficiency of the coating and PRP treatment in terms of homogenous distribution on the synthetic substrate and spreading and overall distribution of cells, respectively. While SEM provided detailed information regarding coatings and cells spreading on restricted areas, micro-CT offers an overall image of the performed modification. As a non-destructive technique, micro-CT is usually used to provide specific architectural information on biological materials or synthetic scaffolds [35]. Micro-CT was also employed by other research groups to evaluate the efficiency of the mineralization process on the polymeric coatings [36,37], but as far as the authors of this study are aware, this technique has never been used to image a thin layer of natural protein coating on a synthetic polymer, probably due to the difficulties of separating two materials with similar X-ray absorbance [38]. Here, we elaborated a protocol which allowed the visualization of the natural polymer coating on the PP mesh, using a staining agent frequently used in histology—silver nitrate. Through fine tuning of the imaging parameters in CTVox we were able to separate the two polymers and visualize only the coatings. The images show an agglomeration of the staining in the knot area of the meshes, stating for a thicker coating in that region. The additional SEM images registered locally in BSED confirmed these findings. In addition to the homogeneity of the coating, the stability is another crucial parameter impacting on the performances of the coated mesh, including the cellular response. Therefore, the assessment of the stability was performed using a biodynamic testing device, under traction and perfusion with PBS. The imaging after the biodynamic treatment showed that the natural polymer is still present on the synthetic meshes, but the coating seems thinner, especially on the length of the meshes filaments. Furthermore, this work provides an in vitro evaluation and comparison of the cellular behavior of L929 seeded on commercially available PP meshes, PP meshes treated with the protein-based hydrogel compositions (GelMA and MuMA, respectively), and their PRP-loaded counterparts. In order to further visualize the cells seeded on the treated meshes, uranyl acetate was used as additional staining. The necessity of a staining agent is well-documented in the specialty literature [39,40] since its absence would have resulted in images in which the scaffold and the cells would appear almost identical. The resulted images offered a map of the cells spreading on the PP meshes. The bright white speckles depicting the cells are present on the whole surface of the mesh. These findings are in good agreement with the quantitative results obtained through LDH. The SEM images show a good adhesion of the cells on the natural coating and provide information on the morphology of the cytoskeleton. As revealed by the LDH assay, the addition of PRP on the PP mesh leads to an increase of approximately 42% in the number of cells at one day post-seeding and approximately 55% at seven days. This data are in agreement with previously investigated PRP-based therapies as recently reviewed [34]. Fibroblast adhesion to PP has been improved when PRP was used as a supplement by Medel et al. for pelvic floor reconstructive surgery [41]. In our study, the hydrogel coating PRP leads to an increase in cell number of over 33% for GelMA and less than 10% for MuMA, providing a method to influence cell response. While a stimulation of cellular interaction was expected for GelMA, the effect of MuMA hydrogel has not been previously described to the best of authors’ knowledge and has further potential for deeper investigation. Whatever the complex mechanisms involved, in can be concluded that the two types of coatings developed in this study provide methods to control the fibroblasts response. The synergistic effect of both naturally-derived protein and PRP concentrate was also demonstrated by this work.

## 5. Conclusions

These results indicate that hydrogel coatings based on GelMA and MuMA have potential to modulate PP mesh integration, while their supplementation with platelet concentrate as a blood-derived bioactivator will further enhance cell interactivity. The most intense scaffold–fibroblasts interactions were obtained when GelMA hydrogel was used and additionally enhanced when GelMA was combined with PRP, suggesting the potential of such coating to stimulate enhanced healing. This may open a new direction in engineering bioactive meshes for hernia repair.

## Supplementary Materials

The following are available online at https://www.mdpi.com/2073-4360/12/8/1677/s1, Figure S1: Micro-CT images and SEM micrographs (insets) at three days post-seeding; left column, top to bottom: PP_3 and protein-coated PP (PPG_3 and PPM_3); right column, top to bottom—PRP-treated PP (PPP_3) and PRP-treated hydrogel-coated sample PPGP_3 and PPMP_3.
