# Supplementary material for: Bioinspired Hydrogel Coating Based on Methacryloyl Gelatin Bioactivates Polypropylene Meshes for Abdominal Wall Repair
Source: Polymers (Basel). 2020 Jul 28;12(8):1677. doi: 10.3390/polym12081677 (PMC7464529; doi:10.3390/polym12081677)
Supplement: Supplementary file 1 [file polymers-12-01677-s001.pdf]

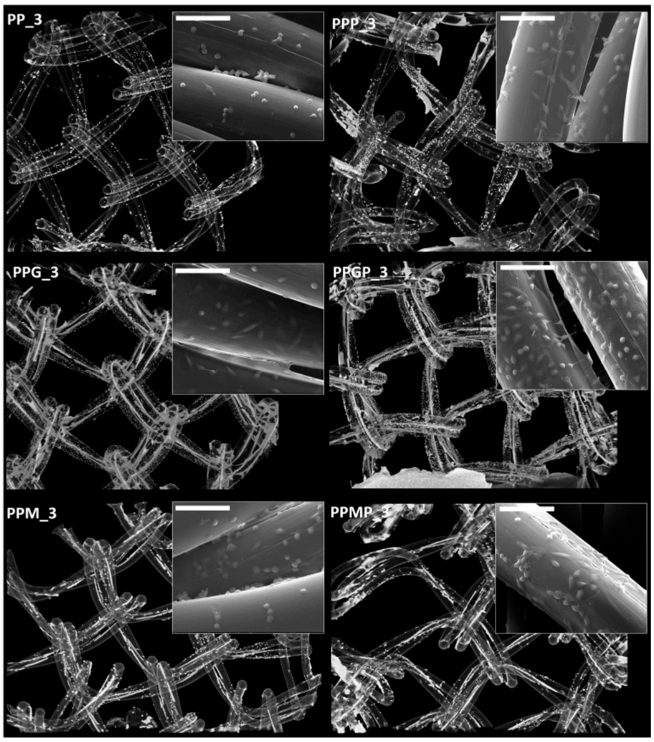

Figure S1. Micro-CT and SEM images at three days post-seeding; left column, top to bottom—PP_3 and protein-coated PP (PPG_3 and PPM_3); right column, top to bottom: PRP-treated PP (PPP_3) and PRP-treated hydrogel-coated sample PPGP_3 and PPMP_3; scalebar 100 μm.
